# Supplementary material for: Diagnostic accuracy of DPP Fever Panel II Asia tests for tropical fever diagnosis
Source: PLoS Negl Trop Dis. 2024 Apr 10;18(4):e0012077. doi: 10.1371/journal.pntd.0012077 (PMC11034646; doi:10.1371/journal.pntd.0012077)
Supplement: S1 Table — (DOCX) [file pntd.0012077.s001.docx]

**S1. Supplementary Table 1.** Reference diagnostic tests.

| **Marker** | **Test** | **Sensitivity &Specificity** |
| --- | --- | --- |
| *Burkholderia pseudomallei* | Blood culture | Sensitivity: 60%  Specificity: 100% |
| *Leptospira,* IgM | SERION ELISA classic Leptospira IgM | Sensitivity: 94.7%  Specificity: >99% |
| *O. tsutsugamushi* IgM | In house ELISA | Sensitivity: 93%  Specificity: 91% |
| *R. typhi* IgM | In house ELISA | Sensitivity: 68%  Specificity: 87.9% |
| Dengue IgM and NS1 | SD Bioline Dengue Duo IgM/IgG/NS1 (CE Marked) | IgG/IgM:   - Sensitivity: 94.2% - Specificity: 96.4%   NS1:   - Sensitivity: 92.4% - Specificity: 94.2% |
| Zika, Chikungunya and Dengue IgM | DPP Zika/ Chikungunya/ Dengue multiplex test (CE-marked) | Sensitivity: 88% to 100% Specificity: >95% |
