# Supplementary material for: Diagnostic accuracy of DPP Fever Panel II Asia tests for tropical fever diagnosis
Source: PLoS Negl Trop Dis. 2024 Apr 10;18(4):e0012077. doi: 10.1371/journal.pntd.0012077 (PMC11034646; doi:10.1371/journal.pntd.0012077)
Supplement: S2 Table — (DOCX) [file pntd.0012077.s002.docx]

**S2. Supplementary Table 2.** Summary statistics for WB assay.

| Micro Reader 1 | Total | Avg. duration of illness (days) | Ref. test positive | DPP test positives | Recommended cut-off value | Sensitivity (%) | Specificity (%) | Optimal cut-off value | Sensitivity (%) | Specificity (%) | AUC value | 95% CI |
| --- | --- | --- | --- | --- | --- | --- | --- | --- | --- | --- | --- | --- |
| *O. tsutsugamushi* IgM | 291 | 7.0 | 21 | 1 | ≥30 | 4.8 | 99.6 | **≥4** | 57.1 | 59.6 | **0.61** | 0.48 - 0.74 |
| *R. typhi* IgM | 291 | 7.6 | 59 | 76 | ≥20 | 62.7 | 83.2 | **≥16** | 76.3 | 73.3 | **0.79** | 0.72 - 0.86 |
| *Leptospira spp.* IgM | 291 | 6.1 | 52 | 122 | ≥20 | 55.8 | 60.7 | **≥21** | 55.8 | 63.6 | **0.60** | 0.51 - 0.70 |
| Dengue IgM | 295 | 5.6 | 36 | 21 | ≥20 | 38.9 | 97.3 | **≥7** | 83.3 | 74.1 | **0.85** | 0.78 - 0.92 |
| Dengue IgG | 295 | 6.9 | 89 | 21 | ≥20 | 14.6 | 96.1 | **≥5.6** | 60.7 | 67.0 | **0.66** | 0.60 - 0.73 |
| Chikungunya IgM | 293 | 5.2 | 14 | 4 | ≥13 | 26.2 | 99.6 | **≥5.3** | 71.4 | 85.0 | **0.82** | 0.67 - 0.95 |
| Zika IgM | 291 | 5.4 | 8 | 0 | ≥20 | 0.0 | 100.0 | **≥3.6** | 100.0 | 84.5 | **0.97** | 0.93 - 1.00 |
| Zika IgG | 285 | 5.8 | 66 | 0 | ≥20 | 0.0 | 100.0 | **≥2.4** | 59.1 | 57.5 | **0.64** | 0.56 - 0.71 |
| Micro Reader 2 |  |  |  |  |  |  |  |  |  |  |  |  |
| *O. tsutsugamushi* IgM | 291 | 7.0 | 21 | 1 | ≥30 | 4.8 | 99.6 | **≥2.9** | 66.7 | 63.0 | **0.71** | 0.62 - 0.79 |
| *R. typhi* IgM | 291 | 7.6 | 59 | 105 | ≥20 | 76.3 | 74.1 | **≥22** | 72.9 | 77.6 | **0.79** | 0.72 - 0.86 |
| *Leptospira spp.* IgM | 291 | 6.1 | 52 | 158 | ≥20 | 59.6 | 46.9 | **≥21** | 57.7 | 50.2 | **0.59** | 0.49 - 0.69 |
| Dengue IgM | 295 | 5.6 | 36 | 24 | ≥20 | 38.9 | 96.1 | **≥8** | 77.8 | 78.0 | **0.84** | 0.77 - 0.92 |
| Dengue IgG | 295 | 6.9 | 89 | 24 | ≥20 | 14.6 | 94.7 | **≥3.8** | 70.8 | 54.9 | **0.68** | 0.61 - 0.75 |
| Chikungunya IgM | 293 | 5.2 | 8 | 8 | ≥13 | 28.6 | 98.3 | **≥4.1** | 78.6 | 78.1 | **0.82** | 0.70 - 0.95 |
| Zika IgM | 291 | 5.4 | 8 | 3 | ≥20 | 37.5 | 100.0 | **≥3.7** | 100.0 | 76.7 | **0.94** | 0.87 - 1.00 |
| Zika IgG | 285 | 5.8 | 66 | 3 | ≥20 | 0.0 | 98.6 | **≥2.5** | 50.0 | 49.3 | **0.51** | 0.42 - 0.59 |
