# Supplementary material for: Diagnostic accuracy of DPP Fever Panel II Asia tests for tropical fever diagnosis
Source: PLoS Negl Trop Dis. 2024 Apr 10;18(4):e0012077. doi: 10.1371/journal.pntd.0012077 (PMC11034646; doi:10.1371/journal.pntd.0012077)
Supplement: S3 Table — (DOCX) [file pntd.0012077.s003.docx]

**S3. Supplementary Table 3.** Summary statistics for serum assay.

| Micro Reader 1 | Total | Avg. duration of illness (days) | Ref. test positive | DPP test positives | Recommended cut-off value | Sensitivity (%) | Specificity (%) | Optimal cut-off value | Sensitivity (%) | Specificity (%) | AUC value | 95% CI |
| --- | --- | --- | --- | --- | --- | --- | --- | --- | --- | --- | --- | --- |
| *O. tsutsugamushi* IgM | 291 | 7.0 | 21 | 3 | ≥30 | 13.1 | 99.3 | **≥4** | 42.9 | 55.2 | **0.49** | 0.34 - 0.65 |
| *R. typhi* IgM | 291 | 7.6 | 59 | 97 | ≥20 | 67.8 | 74.6 | **≥19** | 69.5 | 70.7 | **0.76** | 0.69 - 0.84 |
| *Leptospira spp.* IgM | 291 | 6.1 | 52 | 123 | ≥20 | 48.1 | 58.6 | **≥19** | 50.0 | 54.0 | **0.53** | 0.44 - 0.62 |
| Dengue IgM | 295 | 5.6 | 36 | 27 | ≥20 | 36.1 | 94.6 | **≥9** | 75.0 | 76.8 | **0.81** | 0.73 - 0.90 |
| Dengue IgG | 295 | 6.9 | 89 | 27 | ≥20 | 14.6 | 93.2 | **≥6** | 60.7 | 61.7 | **0.64** | 0.57 - 0.71 |
| Chikungunya IgM | 293 | 5.2 | 14 | 7 | ≥13 | 35.7 | 99.3 | **≥6.1** | 78.6 | 88.2 | **0.86** | 0.72 - 0.99 |
| Zika IgM | 291 | 5.4 | 8 | 1 | ≥20 | 22.0 | 100.0 | **≥4.5** | 87.5 | 90.8 | **0.94** | 0.89 - 1.00 |
| Zika IgG | 285 | 5.8 | 66 | 1 | ≥20 | 0.0 | 99.2 | **≥1.9** | 50.0 | 52.1 | **0.53** | 0.45 - 0.60 |
| Dengue NS1 | 294 | 3.8 | 36 | 55 | ≥20 | 83.3 | 90.3 | **≥25** | 83.3 | 93.4 | **0.88** | 0.80 - 0.97 |
| *B. pseudomallei* CPS Ag | 283 | 10.8 | 8 | 8 | ≥20 | 12.5 | 97.5 | **≥5** | 25.0 | 52.7 | **0.65** | 0.13 - 0.56 |
| Micro Reader 2 |  |  |  |  |  |  |  |  |  |  |  |  |
| *O. tsutsugamushi* IgM | 290 | 7.0 | 21 | 3 | ≥30 | 9.5 | 99.1 | **≥2.7** | 57.1 | 59.1 | **0.59** | 0.45 - 0.72 |
| *R. typhi* IgM | 291 | 7.6 | 59 | 141 | ≥20 | 74.6 | 58.4 | **≥22** | 72.9 | 67.5 | **0.75** | 0.68 - 0.83 |
| *Leptospira spp.* IgM | 290 | 6.1 | 52 | 162 | ≥20 | 57.7 | 44.5 | **≥22** | 51.9 | 52.9 | **0.53** | 0.44 - 0.62 |
| Dengue IgM | 295 | 5.6 | 36 | 38 | ≥20 | 52.8 | 92.7 | **≥8.5** | 80.6 | 74.5 | **0.84** | 0.77 - 0.91 |
| Dengue IgG | 295 | 6.9 | 89 | 38 | ≥20 | 21.4 | 90.8 | **≥5.4** | 60.7 | 58.3 | **0.64** | 0.57 - 0.71 |
| Chikungunya IgM | 293 | 5.2 | 14 | 6 | ≥13 | 35.7 | 98.7 | **≥4.5** | 71.4 | 72.4 | **0.82** | 0.69 - 0.94 |
| Zika IgM | 290 | 5.4 | 8 | 2 | ≥20 | 37.5 | 100.0 | **≥8.3** | 75.0 | 98.6 | **0.91** | 0.79 - 1.00 |
| Zika IgG | 284 | 5.8 | 66 | 2 | ≥20 | 0.0 | 98.6 | **≥3** | 53.9 | 53.4 | **0.53** | 0.44 - 0.61 |
| Dengue NS1 | 294 | 3.8 | 36 | 65 | ≥20 | 88.9 | 87.2 | **≥35** | 86.1 | 93.4 | **0.93** | 0.88 - 0.98 |
| *B. pseudomallei* CPS Ag | 294 | 10.8 | 8 | 11 | ≥20 | 12.5 | 96.4 | **≥6** | 75.0 | 72.4 | **0.71** | 0.29 - 0.67 |
