# Supplementary material for: Diagnostic accuracy of DPP Fever Panel II Asia tests for tropical fever diagnosis
Source: PLoS Negl Trop Dis. 2024 Apr 10;18(4):e0012077. doi: 10.1371/journal.pntd.0012077 (PMC11034646; doi:10.1371/journal.pntd.0012077)

**Supplementary Figure 1.** Estimated accuracies and 95%-confidence intervals for reader performance. Error bars are shown. Legend: x-axis, micro readers 1 and 2 (Micro Reader 1 and 2); light pink circles, WB; green circles, serum.


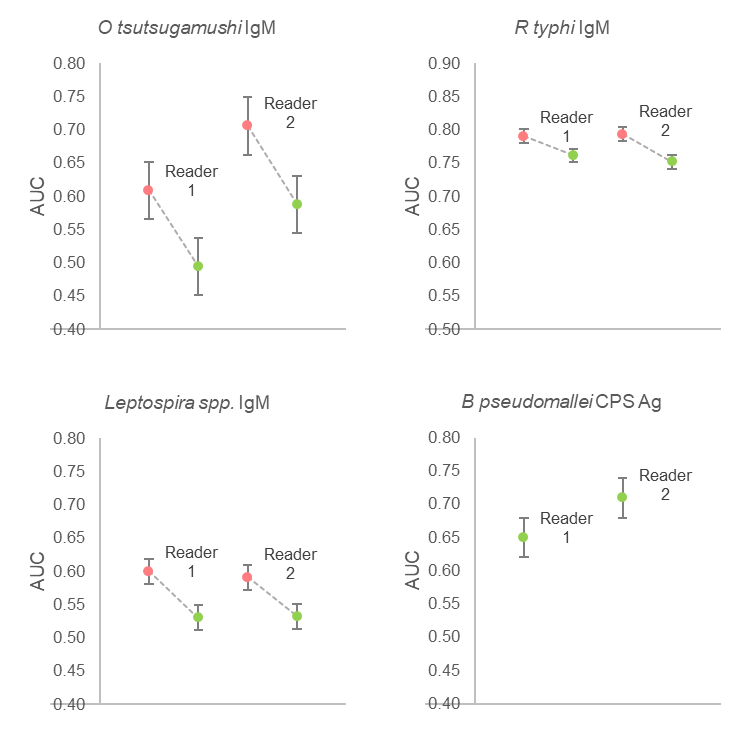


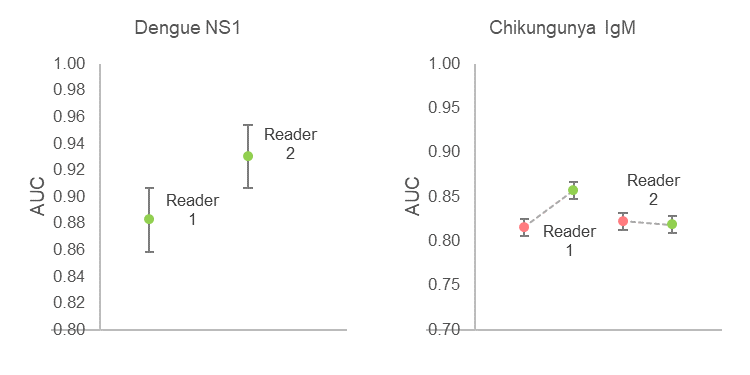


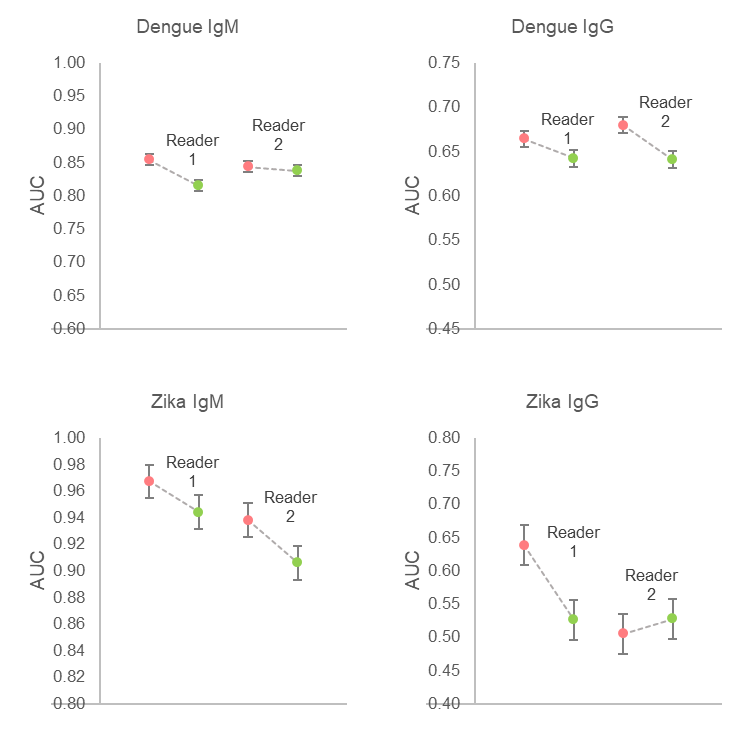

Supplement: S1 Fig — Error bars are shown. Legend: x-axis, micro readers 1 and 2 (Micro Reader 1 and 2); light pink circles, WB; green circles, serum. (DOCX) [file pntd.0012077.s004.docx]
