# Supplementary material for: Diagnostic accuracy of DPP Fever Panel II Asia tests for tropical fever diagnosis
Source: PLoS Negl Trop Dis. 2024 Apr 10;18(4):e0012077. doi: 10.1371/journal.pntd.0012077 (PMC11034646; doi:10.1371/journal.pntd.0012077)
Supplement: Attachment — Submitted filename: PNTD-D-23-00978 Reviewer Comment_SD.docx [file pntd.0012077.s008.docx]

**Reviewer comment: PNTD-D-23-00978**

1. Page 2, Line 44: ….300 patients with undefined illness (UI)… Were these patients with fever? Undefined febrile illness?

Thank you for your comment. The patients did have fever, as such the line has been revised to ‘…300 patients with undefined febrile illness (UFI)’, as suggested.

2. Page 3, Line 51 & Page 37: Supplementary Table 1. Is there any reference study for this in-house R. typhi IgM ELISA? Reference may be provided.

Thank you for your comment. The diagnostic accuracy of the in-house R.typhi ELISA has not yet been published, however, unpublished diagnostic accuracy calculations done by Prof Stuart Blacksell are as follows:

Using the 1:800 IgM IFA cut-off that we calculated for diagnosis of murine typhus infection in Lao patients (i.e., Phakhounthong et al., 2020. The temporal dynamics of humoral immunity to Rickettsia typhi infection in murine typhus patients. Clin Microbiol Infect 2020 Vol. 26 Issue 6 Pages 781 e9-781 e16) the following diagnostic accuracy has been calculated using the following cut-offs for the R typhi IgM ELISA.

0.5 nett OD Sn 68% Sp 87.9%

0.8 nett OD Sn 59% Sp 95%

1.0 nett OD Sn 48.9% Sp 97.7%

As we routinely use a cut-off of 0.5 nett OD, Sn 68% Sp 87.9% have been quoted as the sensitivity and specificity of the test.

3. Page 3, Line 51 & Page 37: Supplementary Table 1. Are these sensitivity and specificity for whole blood as well as serum in the reference studies? If not, sample-specific sensitivity and specificity may be provided in the table.

Thank you for your comment. Reference tests were only done on one sample type – this was the sample type recommended/preferred according to the manufacturer’s instructions.

4. Page 5, Line 103-105: The **lack of non-specific** early presentation confounds diagnosis and subsequent treatment (2, 7). – The lack of specific presentation. Isn’t it?
   Thank you for raising this typographical error. The line has been edited to read ‘the lack of specific presentation…’.
5. Page 7, Line 135-136: the simultaneous detection and differentiation of numerous infections with comparable clinical manifestations. – Although the manifestations are similar, considering the incubation period, the optimal time of detection will differ for different pathogens. How the duration of testing will be optimized for the multiplex RDTs?
   Thank you for your raising this important point. This was addressed in the discussion in lines 388-390: ‘It is recommended to repeat the test after a period of seroconversion to allow for higher confidence in pathogen detection(40).’
6. Page 7, Line 141, 147-148: Manufacturer and country details may be provided.

Thank you for your comment. The details of manufacturer and country have been included in the above-mentioned lines.

7. Page 7-8, Line 152-158: These samples were collected for the UI-study conducted between November 2019 to October 2020. What was the finding of the study? if published reference may be added. The current manuscript is based on leftover specimens from the UI-study. How the specimens were stored and how long the specimens were stored. When was this current method applied?

Thank you for your questions and for raising important details. The UI study conducted is ongoing and is not currently published. The sample storage and collection details have been added to Line 160 – 162, as follows: “Samples used for this study were collected from leftover samples on day samples were received, and were stored at 4C for a maximum of 24 hours prior to testing in this current study.”

8. Page 8, Line 169: Is there any data on failed tests? If so, what is the failure rate?

Thank you for your question. Two invalid test results were recorded, providing a low failure rate of 0.69%. Both failed tests occurred on Micro Reader 1, one serum antibody test and one blood antigen test.

9. Page 8, Line 170: What is the meaning of paired blood and serum specimens? Is the author mentioning test ran parallel in duplicate?

Thank you for your question. Both whole blood and serum from the same patient were tested to compare suitability of sample type. To elucidate this, modifications have been made to Line 173 – 4, to read as follows: “For each patient, testing procedure followed the manufacturer’s instructions and were done with both paired blood and serum specimens (to compare specimen suitability), …”.

10. Page 10, Line 206: At an optimal cut-off where sensitivity and specificity are at a suitable compromise, the DPP® II – It is not clear how the optimal cut-off values of various agents are set/calculated. In methodology, this may be explained with suitable references.
    Thank you for your suggestion. Optimal cut-off values were selected using the results of the ROC curve, and through comparison against the manufacturer’s recommended cut-offs. A clarification of this methodology has been included in Line 204 – 205, reading as follows: ‘A optimal cut-off was selected by maximising both sensitivity and specificity indices from the ROC analysis.’ A suitable reference has also been included, as suggested.
11. Page 15, Line 322: Reason specific - Season-specific serovar

Thank you for your comment. The sentence in Line 322 is meant to suggest that the reason certain region-specific leptospiral serovars cause more severe illness is unknown. For clarification the sentence has been edited as follows: “However, the reason region-specific serovars cause more severe illness remains unknown.”

12. Page 19, Line 400-401: The DPP® Fever Panel II Asia provides quick results without requiring specialized equipment. Are the readers handy and low-cost which is used for interpretation of the result?
13. Thank you for your query. No specialized readers are required for interpretation of results, a digital output is provided on the reader and interpreted using manufacturer’s recommendations. The micro readers are built into the point-of-care test, which is intended for low-resource settings and accordingly cost-effective (the exact price is commercial in confidence with the manufacturer). Lines 405 – 410 clarify that the readers are simple to use and affordable: “In many low-resource settings, where access to diagnostic infrastructure is limited, introducing an adequately sensitive and specific tool would afford immense benefits for point-of-care clinical management and outbreak surveillance. The DPP® Fever Panel II Asia provides quick results without requiring specialised equipment. Given the ease with which the test can be performed, it serves both clinical and field utility, especially when health workers may have limited training(40).”
14. The authors should state how the samples used as "true positives" were confirmed to be so. Was it based on clinical presentation, some other diagnostic test or a combination of both? When developing and testing a new assay it is crucial that the "known positives" are indeed "genuine true positives".

Thank you for raising this important point. As all patients presented with undefined fever, clinical presentation alone did not provide a confirmation – true positives were determined as reference test positives. This clarification has been included to the Methods, line 188: “True positives were determined as positives by reference diagnostic tests”, as per the reviewer’s recommendations.

This limitation was also previously addressed in the Discussion, lines 389-390 as follows: “The main limitations of the study are the restricted sample size it was conducted in; an absence of true positives (Table 1) can bias sensitivity and specificity, which does not parallel real-life settings.” The following lines 391-393, were revised to emphasise the limitations of the test: “The diagnostic accuracy of the DPP® Fever Panel II Asia was overall limited, while the sensitivity of the diagnostic panel is lower than the specificity, it is likely attributed to low levels of antibody during the acute phase of infection(51).”

15. What is the "CPS" antigen of B.pseudomallei ? Some readers (including me) may not know what it is!

Thank you for your comment. I have now addressed this in the Introduction, lines 138-140, which has been revised to read as follows: “Quantitative readings for specific antigens can also serve as indices of severity, as has been shown for histidine-rich protein 2 (HRP2) in malaria(22), capsular polysaccharide (CPS) in melioidosis(23), and non-structural protein 1 (NS1) in dengue(24).”
