# Supplementary material for: Diagnostic accuracy of DPP Fever Panel II Asia tests for tropical fever diagnosis
Source: PLoS Negl Trop Dis. 2024 Apr 10;18(4):e0012077. doi: 10.1371/journal.pntd.0012077 (PMC11034646; doi:10.1371/journal.pntd.0012077)
Supplement: Attachment — Submitted filename: PNTD-D-23-00978 Reviewer Comment.docx [file pntd.0012077.s006.docx]

**Reviewer comment: PNTD-D-23-00978**

1. Page 2, Line 44: ….300 patients with undefined illness (UI)… Were these patients with fever? Undefined febrile illness?
2. Page 3, Line 51 & Page 37: Supplementary Table 1. Is there any reference study for this in-house R. typhi IgM ELISA? Reference may be provided.
3. Page 3, Line 51 & Page 37: Supplementary Table 1. Are these sensitivity and specificity for whole blood as well as serum in the reference studies? If not, sample-specific sensitivity and specificity may be provided in the table.
4. Page 5, Line 103-105: The **lack of non-specific** early presentation confounds diagnosis and subsequent treatment (2, 7). – The lack of specific presentation. Isn’t it?
5. Page 7, Line 135-136: the simultaneous detection and differentiation of numerous infections with comparable clinical manifestations. –
6. Although the manifestations are similar, considering the incubation period, the optimal time of detection will differ for different pathogens. How the duration of testing will be optimized for the multiplex RDTs?
7. Page 7, Line 141, 147-148: Manufacturer and country details may be provided.
8. Page 7-8, Line 152-158: These samples were collected for the UI-study conducted between November 2019 to October 2020. What was the finding of the study? if published reference may be added. The current manuscript is based on leftover specimens from the UI-study. How the specimens were stored and how long the specimens were stored. When was this current method applied?
9. Page 8, Line 169: Is there any data on failed tests? If so, what is the failure rate?
10. Page 8, Line 170: What is the meaning of paired blood and serum specimens? Is the author mentioning test ran parallel in duplicate?
11. Page 10, Line 206: At an optimal cut-off where sensitivity and specificity are at a suitable compromise, the DPP® II
12. It is not clear how the optimal cut-off values of various agents are set/calculated. In methodology, this may be explained with suitable references.
13. Page 15, Line 322: Reason specific - Season-specific serovar
14. Page 19, Line 400-401: The DPP® Fever Panel II Asia provides quick results without requiring specialized equipment. Are the readers handy and low-cost which is used for interpretation of the result?
